# Thanks to all those who reviewed for *Biological Procedures Online* in 2014

**DOI:** 10.1186/s12575-015-0017-7

**Published:** 2015-01-31

**Authors:** Shulin Li

**Affiliations:** Department of Pediatrics–Research, Unit 0853, The University of Texas MD Anderson Cancer Center, 1515 Holcombe Blvd., Houston, TX 77030 USA

## Abstract

A peer-reviewed journal would not survive without the generous time and insightful comments of the reviewers, whose efforts often go unrecognized. Although final decisions are always editorial, they are greatly facilitated by the deeper technical knowledge, scientific insights, understanding of social consequences, and passion that reviewers bring to our deliberations. For these reasons, the Editor-in-Chief and staff of the journal warmly thank the reviewers whose comments helped to shape *Biological Procedures Online,* for their invaluable assistance with review of manuscripts for the journal in Volume 16 (2014).

**Aruna Ambagala**

Canada

**Hosam Arafate**

Saudi Arabia

**Deb Court**

Canada

**Marina Cretich**

Italy

**Wim Dhaeze**

USA

**Marxa Figueiredo**

USA

**Ching-Hung Hsu**

USA

**Ching-Hung Hsu**

China

**Yingqi Hua**

USA

**Michael Hust**

Germany

**David le Couteur**

Australia

**Bolin Liu**

USA

**Lucia Manso-Silvan**

France

**Nitish Mishra**

USA

**Mauro Provinciali**

Italy

**Sebastien Rodrigue**

Canada

**Lucia Sacchetti**

Italy

**Akinori Sato**

Japan

**Masatoshi Tagawa**

Japan

**Lynn Thomason**

USA

**Shi Xu**

USA

**Shaomian Yao**

USA

